# New insights into the biology and development of lung cancer in never smokers—implications for early detection and treatment

**DOI:** 10.1186/s12967-023-04430-x

**Published:** 2023-08-31

**Authors:** Peiyao Wang, Sophie Sun, Stephen Lam, William W. Lockwood

**Affiliations:** 1 Department of Integrative Oncology, British Columbia Cancer Research Institute, Vancouver, BC Canada; 2 Interdisciplinary Oncology Program, University of British Columbia, Vancouver, BC Canada; 3 Department of Medical Oncology, British Columbia Cancer Agency Vancouver, Vancouver, BC Canada; 4 Department of Pathology and Laboratory Medicine, University of British Columbia, Vancouver, BC Canada

**Keywords:** Multi-omics, Genomics, Lung adenocarcinoma, Never smokers, Lung cancer in never smokers

## Abstract

Lung cancer is the leading cause of cancer deaths worldwide. Despite never smokers comprising between 10 and 25% of all cases, lung cancer in never smokers (LCNS) is relatively under characterized from an etiological and biological perspective. The application of multi-omics techniques on large patient cohorts has significantly advanced the current understanding of LCNS tumor biology. By synthesizing the findings of multi-omics studies on LCNS from a clinical perspective, we can directly translate knowledge regarding tumor biology into implications for patient care. Primarily focused on never smokers with lung adenocarcinoma, this review details the predominance of driver mutations, particularly in East Asian patients, as well as the frequency and importance of germline variants in LCNS. The mutational patterns present in LCNS tumors are thoroughly explored, highlighting the high abundance of the APOBEC signature. Moreover, this review recognizes the spectrum of immune profiles present in LCNS tumors and posits how it can be translated to treatment selection. The recurring and novel insights from multi-omics studies on LCNS tumor biology have a wide range of clinical implications. Risk factors such as exposure to outdoor air pollution, second hand smoke, and potentially diet have a genomic imprint in LCNS at varying degrees, and although they do not encompass all LCNS cases, they can be leveraged to stratify risk. Germline variants similarly contribute to a notable proportion of LCNS, which warrants detailed documentation of family history of lung cancer among never smokers and demonstrates value in developing testing for pathogenic variants in never smokers for early detection in the future. Molecular driver subtypes and specific co-mutations and mutational signatures have prognostic value in LCNS and can guide treatment selection. LCNS tumors with no known driver alterations tend to be stem-like and genes contributing to this state may serve as potential therapeutic targets. Overall, the comprehensive findings of multi-omics studies exert a wide influence on clinical management and future research directions in the realm of LCNS.

## Background

**Fig. 1:**
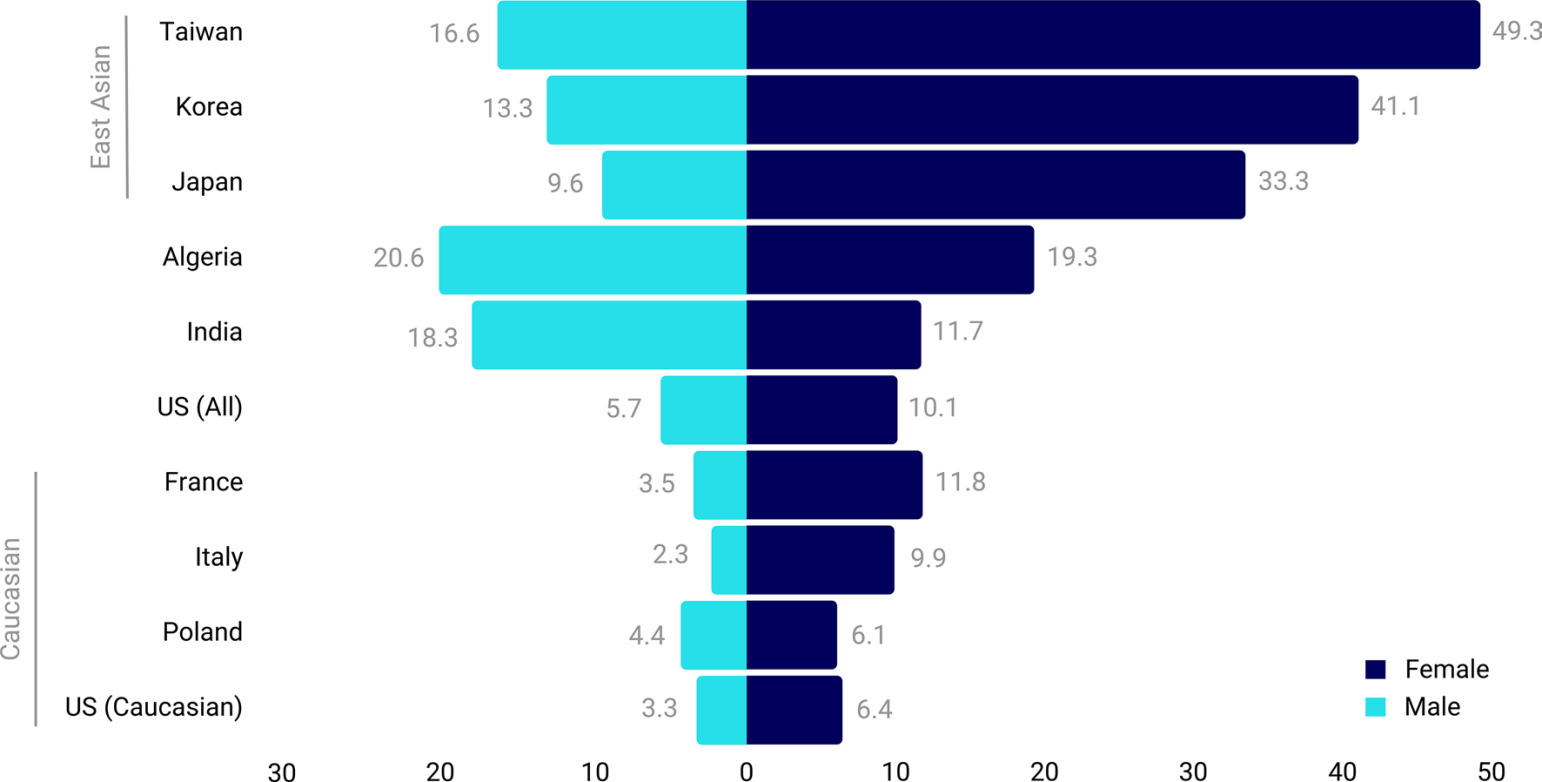
Global variation in proportions of lung adenocarcinoma patients who are never smokers stratified by sex. Data from studies working with national and regional hospital registries that explicitly reported lung adenocarcinoma or lung cancer histology, smoking status, and sex [11–19]. *US *United States

Lung cancer in never smokers (LCNS) is relatively under characterized from an etiological and biological perspective, despite comprising 10–25% of all lung cancer cases [1]. Defined as individuals with a lifetime smoking history of less than 100 tobacco cigarettes, LCNS as an independent disease would constitute the seventh leading cause of cancer-related deaths globally [2]. Commonly presenting as non-small cell lung cancer (NSCLC), adenocarcinoma subtype (LUAD), the burden of LCNS has been climbing worldwide as the proportion of LUAD cases in never smokers relative to total lung cancer cases has been consistently increasing for both sexes, independent of geographical region, since the 1950s [3, 4]. Despite the global rise in cases of LUAD among never smokers, there is a wide range in prevalence and distribution based on sex and geographic location that remains unexplained. Specifically, females of East Asian descent account for the majority of lung cancer cases in never smokers, whereas males of Caucasian descent are less likely to be diagnosed with LCNS (Fig. 1) [5].

The understanding of LCNS carcinogenesis as well as the assessment of factors influencing its risk of development is incomplete. Many observational studies have explored different factors associated with LCNS and have yielded varied results. Potential risk factors have been studied, including genetic predisposition, second hand smoke, radon exposure, outdoor air pollution, diagnosis of COPD, conditions related to immune system and previous infections [6–9]. The majority of these risk factors have shown to potentially contribute to LCNS to some extent; however, there remains a significant proportion of LCNS cases that are not been associated with any known risk factors or exposures [10]. This suggests that not only is LCNS a distinct entity from smoking lung cancer, but in itself, it is a clinically and molecularly heterogeneous disease.

In recent years, with the surge of research interest in LCNS, a niche for investigating LCNS through integrative global genomic and transcriptomic techniques has emerged. This provides the opportunity to empirically probe for patterns in LCNS tumor biology that can account for the observed wide clinical variation, which can subsequently direct both pre-clinical and clinical research to improve LCNS detection and treatment.

This review describes the multi-omic and biological landscape of LCNS uncovered by recent integrative genomic analyses using large-scale tumor cohorts. Primarily focused on never smokers with LUAD, the driver mutations, mutational patterns, germline variants, and immune profiles of their tumor samples will be detailed. The aim of synthesizing these results is to shed light on potential early detection and treatment strategies for LCNS and the utility of genomics in the clinical care of LCNS patients.

## Methodology

The main articles of focus for this review were retrieved from PubMed using the keywords “NSCLC”, “never smoker”, and “genomics” with their respective alternative terms from January 1, 2015 to March 1 2023. Among available studies of never smoking patients with NSCLC, there have been three seminal publications that integrate multiple sequencing methodologies to study underlying tumor biology of the disease [20–22]. These studies are transformative to the field of LCNS research in that they provide a biology driven view of the etiology and nature of LCNS.

These are also the only studies of their kind that each comprise of over 50 LCNS tumor samples and employ more than one next generation sequencing method. The first cohort from Devarakonda et al. included 160 total never smoker LUAD patients, whose tumors were studied via whole exome sequencing and RNA sequencing. The second cohort from Zhang et al. consisted of 232 never smoking NSCLC patients whose tumors were analyzed via whole genome sequencing and RNA sequencing. Lastly, a third cohort from Chen et al. involved whole exome sequencing and RNA sequencing in addition to proteome and phosphoproteome analysis of tissue samples from 103 patients, of which 85 were never smokers. Age, ethnicity, and sex varied across these the studies and are further detailed in Table 1.

**Table 1:** Clinical characteristics and multi-omics methods of highlighted articles

	Devarakonda et al. [20]	Zhang et al. [22]	Chen et al. [21]
Sample size for genomic analysis	459	232	103
Number of never smokers	88 (+ 76 NS external samples)	232	85
Sample type	Lung tumor, adjacent normal tissue	Lung tumor, adjacent normal tissue, and blood	Lung tumor, adjacent normal tissue, and blood
NSCLC Type	LUAD (100%)	LUAD (81.5%) Carcinoids (15.5%) Other (3%)	LUAD (100%)
Mean patient age at diagnosis (SD)	68.2 (11.8)	64.8 (11.9)	63.5 (10.3)
Patient sex (%)	Male (29.9%)	Male (24.6%)	Male (29.4%)
	Female (70.1%)	Female (75.4%)	Female (70.6%)
Never smoker patient ethnicity (%)	Caucasian (72.7%)	European (97%)	Taiwanese (100%)
	Asian (12.5%)	Asian (2%)	
	African (9.1%)	African (1%)	
	Other (5.7%)		
Methods	Whole exome sequencing	Whole genome sequencing	Whole exome sequencing
			RNA sequencing
	RNA sequencing	35 LUAD underwent RNA-Seq	Proteome analysis
			Phosphoproteome analysis

## Molecular alterations

**Fig. 2:**
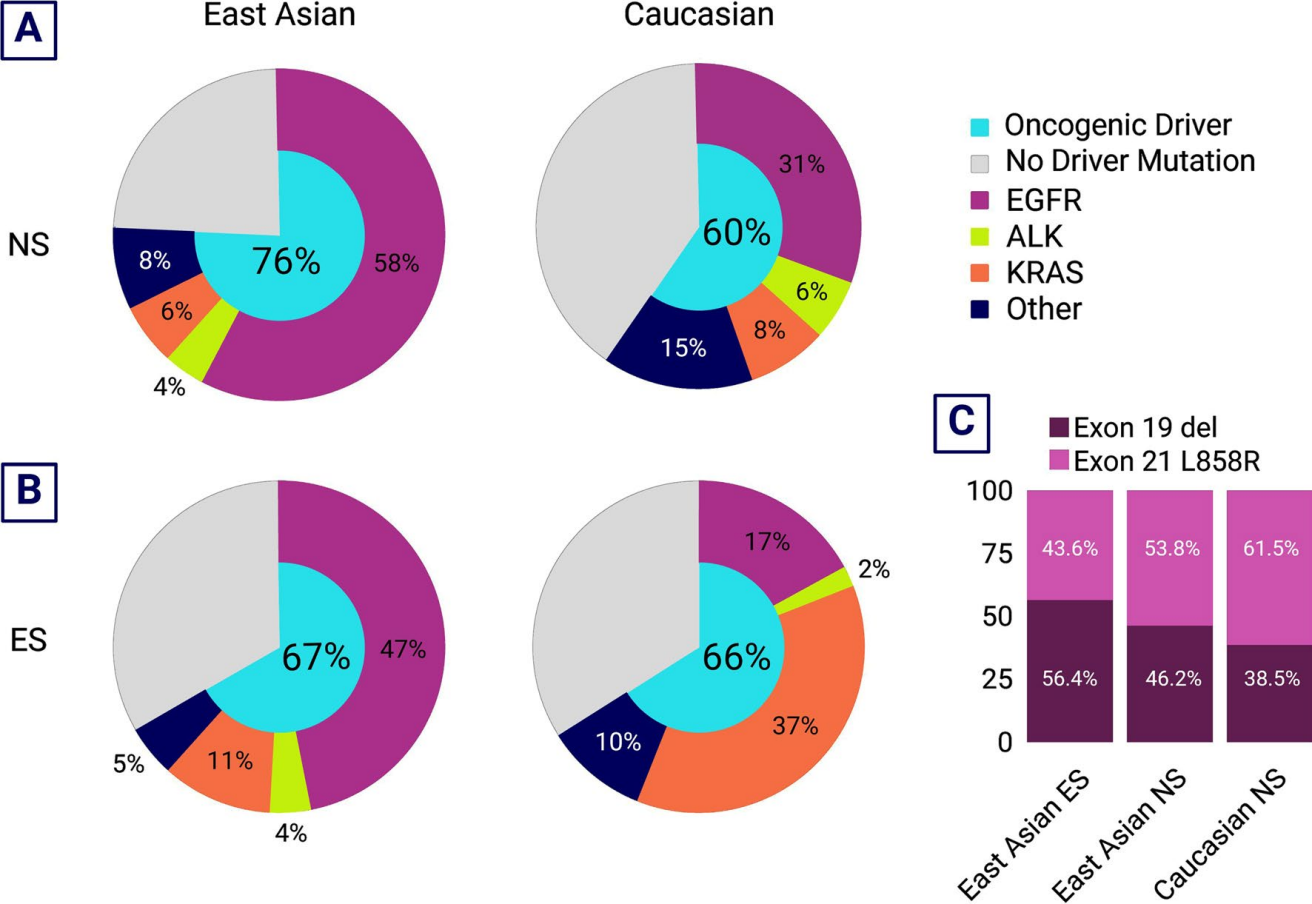
Oncogenic drivers in lung cancer patients based on smoking status and ethnicity. **A** Distribution of oncogenic drivers in East Asian (n = 484) [24, 26, 27] and Caucasian (n = 264) [22, 28] never smoker lung cancer patients (majority with lung adenocarcinoma). **B** Distribution of oncogenic drivers in East Asian (n = 248) [27, 29] and Caucasian (n = 7187) [28, 30] ever smoker lung cancer patients (majority with lung adenocarcinoma). **C** Ratio of EGFR exon 19 deletion to exon 21 L858R substitution between East Asian ever smokers (n = 39) [31], East Asian never smokers (Taiwanese, n = 65) [21], and Caucasian never smokers (n = 39) [20]. *NS *never smoker; *ES *ever smoker

Targetable molecular driver mutations are significantly more common in LCNS as compared to their smoking counterparts, making them great candidates for targeted therapies [23]. Genomic analyses of the three cohorts show that a relatively high proportion of LCNS patients harbor mutations in driver genes, particularly in EGFR, consistent with prior studies comparing never smokers versus smokers with lung cancer [20, 22]. RTK/RAS/RAF pathway driver alterations are also commonly detected in LCNS, are often mutually exclusive, and occur more frequently in never smokers as compared to smokers among patients of the same ethnicity (Fig. 2A, B) [20, 22]. However, the overall frequency of oncogenic driver mutations varies based on ethnicity, ranging from 60% among Caucasian never smokers as compared to 76% among patients of East Asian ancestry (Fig. 2A) [22, 24].

This difference is greater when focusing on the frequency of EGFR mutations among cohorts of non-Asian cohorts predominantly of European descent. For example, Zhang et al. reported 30.6% of cases having an EGFR mutation in their almost entirely Caucasian cohort, while Chen et al. reported an 87% EGFR mutation detection rate in their never smoking Taiwanese sub-cohort. In comparison, Devarakonda et al. identified a mix of different ethnicities, of which 52.3% of patients were EGFR mutation positive.

Among sensitizing EGFR mutation subtypes, 33.4% were EGFR exon 19 deletions in the Devarakonda et al. study, and the majority of the cohort (73%) were Caucasian. In comparison, 40.5% of EGFR mutated LCNS tumors in the Taiwanese cohort had the same mutation (Fig. 2C). Previous comparisons between smoker and never smoker LUAD patients have found no differences in EGFR mutation subtype frequency [25], although comparison of EGFR sub-mutations across ethnicities within LCNS patient populations has not been performed previously. Although no significant differences appear to be present between a Taiwanese and predominantly Caucasian cohort, additional investigation is needed to potentially inform treatment approaches for different LCNS populations.

The unique subset of EGFR mutation-lacking LCNS patients in Caucasian populations coincides with a subset of LCNS tumors that have been identified to have lower tumor mutational burden (TMB) and lack somatic copy number alterations (SCNAs), structural variants, and whole genome doubling as reported by Zhang et al., termed the ‘piano’ subtype. This group of 115 cases (49.5% of the cohort), of which 78 were LUAD, generally lacked dominant driver alterations with the exception of KRAS-76.5% of the KRAS-mutated tumors fell into this subtype. The other main driver mutations found in this subtype, RET fusion as well as mutations in NKX2-1, which regulates RET, were present in a small proportion and exclusive to piano tumors. This implicates that these patients have limited available targeted therapy treatment options. However, the ‘stemmness’ of these tumors suggests potential for the development of future therapies that target mutations identified in NOTCH1 or ARID1A pathway within the cohort from Zhang et al. These genes are involved in the initial differentiation of progenitor cells and thus potentially tumor cell differentiation [32, 33]. In contrast, cases with the highest SCNAs and TMB were most likely to have TP53 mutations or TP53 mutations co-occurring with EGFR mutations. In addition to TP53, EGFR mutations were found to significantly co-occur with CDKN2A and RB1 in LCNS, which have potential therapeutic and prognostic value, respectively [20].

EGFR-mutated tumors present their most recent common ancestor (MRCA), a cell containing all the alterations that will lead to carcinogenesis, around the age of 61, which is a median of eight years before the tumor becomes clinically evident [22]. Determined within tumor tissue by measuring mutations that are known to occur at a steady rate from a previously defined model [22], this requires further validation but implies a sizeable window of time for potential EGFR mutation screening and its co-occurring alterations. Similarly, stem-like piano subtype tumors have a median latency of 9.1 years, presenting another opportunity for early detection if characteristics of this population can be better defined.

## Proteomic trends

Chen et al. investigated proteomic trends within LCNS tumors and explored how this data correlated with genomic and transcriptomic findings. Clustering the tumors by proteomic profile led to three subgroups that were separated by tumor staging. This division by disease stage was not present when clustering RNA profiles within the cohort, demonstrating the value of using multi-omic methods to understand LCNS tumor biology. The proteomic subdivisions of the patient cohort also coincided with driver mutations and genomic characteristics, clustering late-stage tumors with TP53 mutation and relatively high TMB and also early stage patients who specifically lacked EGFR L858R substitutions [21].

Six members of the APOBEC3 protein family that are associated with APOBEC mutagenesis were found in 30% of Chen et al.’s cohort and present at higher levels in females than males on the proteomic level but not the RNA level. Females with high APOBEC mutational signatures had higher expression of kinases CK2, CDK1, and CDK2 than in other LCNS patients, suggesting specific treatment regimens that may have better outcomes in this group.

## Mutational signatures

Mutational signatures are a critical component of understanding the underlying processes involved in carcinogenesis. These signatures consist of somatic mutations, including substitutions and copy number variations, that are consistently observed in certain processes [34, 35]. While some mutational processes are exogenous, resulting from exposure to factors such as tobacco smoke or UV radiation, others are endogenous, arising from mutagenic processes related to aging or inflammation, such as APOBEC cytosine deaminase mediated DNA damage [35, 36].

**Fig. 3:**
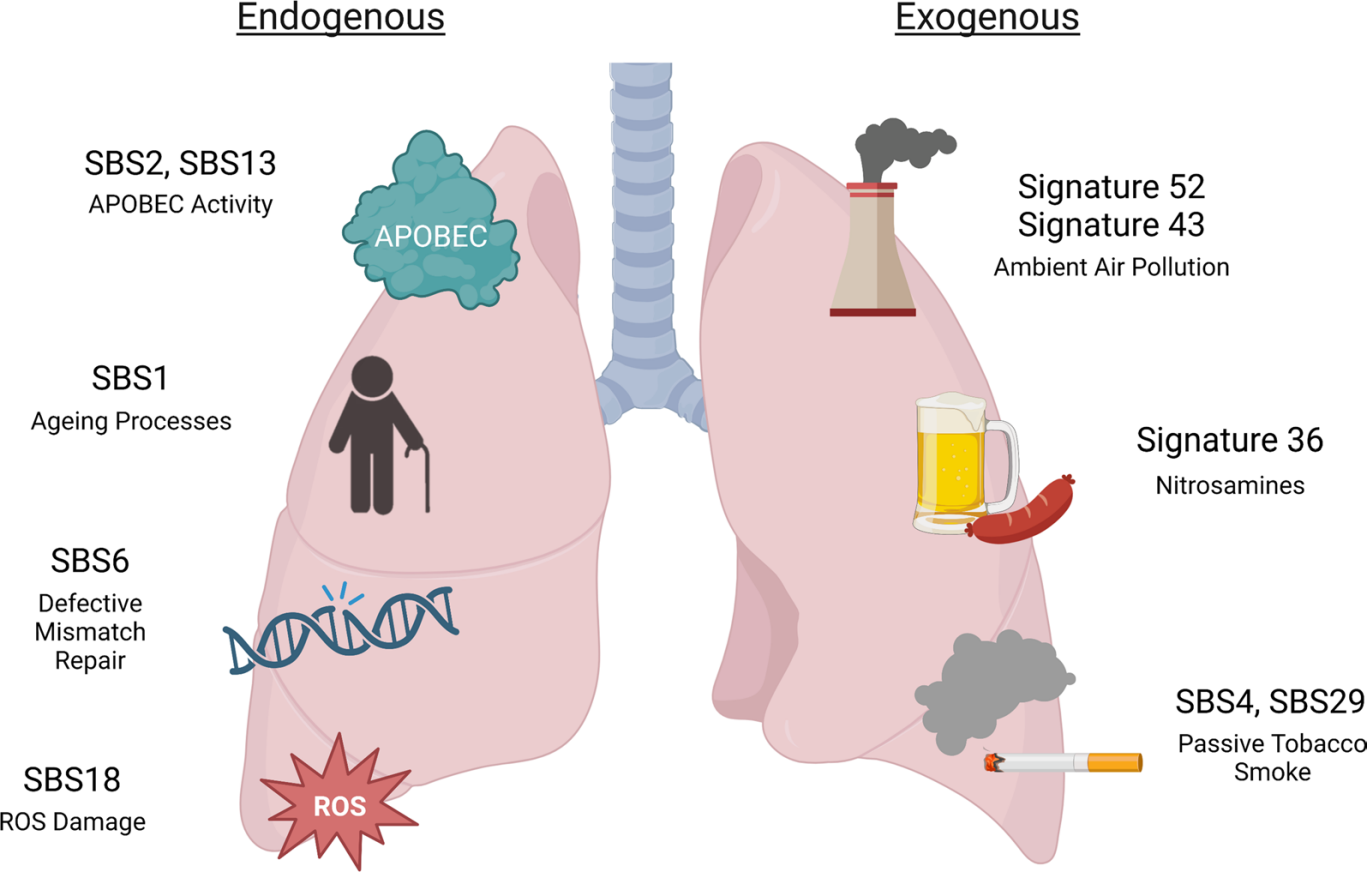
Mutational signatures identified within lung cancer in never smoker tumors. The depicted mutational signatures shown are associated with defined endogenous and exogenous etiologies. SBS5, SBS8, and SBS16, SBS40 have also been reported in LCNS but their etiology remains unknown. SBS = single base substitution

A wide range of both exogenous and endogenous mutational signatures has been observed in LCNS tumors (Fig. 3). Understanding mutational signatures is not only important for unraveling the etiology of diseases but also has significant implications to inform clinical management. For example, mutational signatures can be used to predict treatment response and provide prognosis [37]. In the case of tobacco smoking related lung cancer, mutational signatures have been shown to be predictive of treatment response and can guide patient management [38, 39]. With the increasing availability of whole exome and whole genome sequencing, understanding mutational signatures within diseases like LCNS tumors holds great promise for improving patient outcomes.

### Exogenous

#### Second hand smoke

Second hand tobacco smoke (SHS) exposure is reflected in only a small proportion of LCNS tumor biology, diminishing the role that SHS has previously been believed to have in LCNS tumor carcinogenesis [40, 41]. In these three cohorts, tobacco smoke related mutational patterns are much less frequent than in actively smoking lung cancer patients. Single base substitution 4 (SBS4) is a COSMIC mutational signature that is commonly observed in lung cancer tumors of currently tobacco smoking patients, characterized by C> A substitutions resulting from tobacco mutagen exposure [42]. In a subset of 62 never smoking lung cancer patients reporting SHS exposure, Zhang et al. revealed the absence of SBS4 signatures or related signatures. This outcome is consistent with a Belgian study that also found no evidence of SBS4 in its 46 patient LCNS cohort [43]. This suggests that SHS exposure may trigger a mutational signature distinct from SBS4 or may influence lung cancer development independently of mutational patterns.

Conversely, three of the five mutational profiles identified among the Taiwanese cohort of LCNS patients harbored mutational signatures that are related to tobacco mutagens [21]. Two of these profiles contained SBS4 and the third included SBS29, which is related to chewing tobacco mutagens. All three signatures had high similarity with mutational signatures that are characteristic of outdoor air pollution such as nitrated polycyclic aromatic hydrocarbons (nitro-PAHs) and PAHs [21]. Thus, in the subset of LCNS patients in which SHS plays a role, there may be shared or synergistic effects of SHS and outdoor air pollution, leading to similar mutational patterns.

Furthermore, Devarakonda et al. detected a cigarette smoke mutagen signature in the form of SBS29 in 5.9% (n = 9) of LCNS patients, indicating a possible independent role for passive exposure to cigarette smoke in a small subset of LCNS cases [20]. Therefore, the impact of SHS history may be contingent on the home environment of the population studied, and at most plays a minor role in LCNS incidence.

#### Outdoor air pollution

Outdoor air pollution may play a greater role in LCNS risk than previously postulated, with genomic impact of its exposure reported in all three cohorts. In a primarily European cohort, Zhang et al. found six tumors with a nitro-PAH mutational signature, known as signature 52 in the compendium generated by Kucab et al. [44], which accounted for 18.7% of the single nucleotide variants in the overall cohort. Nitro-PAHs and PAHs are formed from incomplete combustion of fossil fuels and biomass and thus potent sources of emission include vehicles, industrial processes, and forest fires [45]. Regarded as a carcinogenic air pollutant, nitro-PAH can be present in very fine particles down to the size of < 1 µm, which can then accumulate in the distal airways over time [46]. The same nitro-PAH signature along with other PAH mutational signatures were also present in 84% of the LCNS patients of the institutional cohort of Devarakonda et al.

The mutational profiles identified in the Taiwanese cohort by Chen et al. provide additional insight into the distinct impact of outdoor air pollution in the LCNS population, potentially due to higher rates of exposure in Asia compared to Europe. Signature 52 and signature 43, representative of nitro-PAH and PAH exposure respectively, were uniquely enriched in this cohort, comprising two of the five mutational profiles identified [44]. The nitro-PAH signature was found to be overrepresented in older females and those with EGFR mutations [21], suggesting a possible increased vulnerability to LCNS from outdoor air pollution that correlates with years of exposure. There are multiple mechanisms through which outdoor air pollution may drive lung carcinogenesis. One such mechanism is that ambient fine particulate matter exposure leads to accumulation of DNA damage in the lung that translate to both the protein and mRNA level and is irreparable over time, similar to carcinogenesis from UV light and radiation [47]. Another recent study has posited that outdoor air pollution exposure selects for proliferation of pre-existing cells with EGFR mutation, also providing a mechanism by which EGFR mutation rate is higher in the tumors of those living in areas with relatively higher levels of air pollution [48]. The increasing recognition of the importance of ambient air pollution in LCNS highlights the need for further research to optimize treatments and improve survival in patients harboring these signatures.

#### Diet

Interestingly, a third mutational profile defined by Chen et al. involves a signature representative of N-nitrosopyrrolidine or nitrosamine-like compounds, commonly found in tobacco as well as foods such as cured meats, bacon, beer, and whiskey [49, 50]. This may be the first reported genomic evidence of diet possibly impacting LCNS carcinogenesis. Previous evidence of the role of diet in lung cancer has been modest, particularly in the context of never smokers. Observational studies have found that high red meat consumption may increase lung cancer incidence across several populations [51]. A proposed mechanism for this has been the formation of carcinogenic compounds like PAHs and nitrosamines formed in the high temperature cooking of red meat [51], which may be corroborate the presence nitrosamine-like mutational signatures in a subset of LCNS patients.

Overlaying the mutational signatures on chromosomal regions, Chen et al. found that nitro-PAH and nitrosamine-like signatures were enriched in chromosome 7p, the location of EGFR, and the nitro-PAH signature was enriched at the chromosomal region of TP53 [21]. Combining these findings with higher outdoor air pollution levels in East Asia compared to other geographic locations suggests a potential process by which East Asian populations have higher rates of EGFR alterations than other populations.

### Endogenous

#### APOBEC activity

Endogenous signatures in LCNS tumors have potential diagnostic and therapeutic implications. SBS2 and SBS13 mutational signatures, which are associated with the AID/APOBEC activity of cytidine deaminases and innate immune response, are frequently detected in LCNS cohorts and as well as in a variety of other cancer cohorts including within smoking lung cancer [39, 52, 53].

In the study by Chen et al., the APOBEC signature was found in 44% of LCNS patients, with 74% of cases presenting in younger females (≤ 60) and 100% of females without an activating EGFR mutation. Conversely, 95% of cases lacking APOBEC signatures had an EGFR mutation, implying an incompatible relationship between APOBEC signatures and EGFR mutation in females from this cohort [21]. On the other hand, Zhang et al. reported a strong correlation between APOBEC signature and RTK/RAS/RAF positive tumors as well as TP53 mutations [22]. However, the heterogeneity of the tumors with APOBEC signature in this cohort made it difficult to interpret any associations regarding APOBEC signature co-occurring with EGFR mutation and within specific ethnicities [22].

#### Ageing and unknown etiologies

In addition to SBS2 and SBS13 also being the predominant mutation signatures in the Devarakonda et al. cohort, SBS1 and SBS6 were frequently reported as well. These signatures are associated with endogenous pathways and have been correlated with ageing and mismatch repair deficient tumors, respectively. Other mutational signatures such as SBS5, 8, and 40 were also detected in Zhang et al., albeit not prominently and their etiologies remain largely unknown. Moreover, a Belgian study of LCNS patients found SBS16 as the most common signature, present within 31% of patients, which is unique from all previously mentioned cohorts [43]. These findings underscore the diversity across LCNS tumors and substantiates the need to consider geographic locations, ethnicities, environmental exposures and diet when evaluating genomic patterns.

#### ROS damage

Damage from reactive oxygen species (ROS) may be another pathway linked to LCNS carcinogenesis. Attributing to SBS18, 46% of samples in the study by Zhang et al. had this mutational signature, with a preferential occurrence in tumors with EGFR mutations and higher TMB [22]. Although not previously explored in the context of LCNS, some of these endogenous signatures have been tied to propensity for metastasis and preferential response to different therapies, so further research in this area may provide valuable guidance to LCNS management.

## Germline variants

Germline variants may confer a greater risk of LCNS and identifying at-risk individuals and families with inherited cancer predisposition genes can potentially inform screening and early detection. Known pathogenic germline variants have been reported among LCNS patients. Devarakonda et al. found that 6.9% of never smokers with lung cancer in their study had a pathogenic or likely pathogenic germline variant, some of which were in cancer-associated genes such as *FANCG* and *TMEM127* [20]. Although this percentage was not significantly different from smoker cases with LUAD, certain cancer predisposition genes were observed only in never smoker patients [20]. Among the cancer-related germline variants exclusive to never smokers, *BRCA1*, *MSH6*, and *NF1* were also detected in the LCNS cases from Zhang et al., with most of these patients having a piano subtype or stem-like mutational profile that lacked driver mutations [20, 22]. The cohort from Zhang et al. reported 36.6% (85/232) cases with pathogenic or likely pathogenic germline variants, although the threshold employed for pathogenic variant detection was much lower than that of Devarakonda et al. In addition, both of these cohorts are of mainly European descent; a genome-wide association studies (GWAS) of LUAD in an East Asian cohort proposed 25 independent loci that conferred higher risk of LUAD that was more strongly associated with never smokers than smokers, but the results did not transfer to LUAD patients of European descent [54].

Germline analysis also suggests that hormones play a role in LCNS pathogenesis, as Zhang et al. observed repeated germline variants in CYP21A2 (n = 8), involved in cortisol and aldosterone synthesis, and AR (n = 5), encoding the androgen receptor [22]. As hormonal circulation varies greatly between sexes, this may provide a genomic basis for the significantly higher prevalence of LCNS in females than in males [55].

Additionally, somatic alterations occur independently of germline variants as determined by Devarakonda et al., who detected no relationship between germline variants and somatic alterations in genes common to LUAD. Thus, investigation of both may be required for adequate genomic assessment of the patient [20]. These findings indicate the potential of germline variants in identifying high risk LCNS patients, with stratification by sex and ethnicity holding additional promise for improved risk assessment.

## Immune profile

Although LCNS patients have poorer responses to immunotherapy than those of smoking lung cancer patients, there are varied immune profiles among LCNS tumors and the clinical implications of this remain unclear. In early-stage (primarily IA) LCNS tumors, immune cell transcription factors involved in lymphoid cell activation were found to be upregulated, correlating with the expression of proteins predominantly found in B cells, T cells, and NK cells [21]. High concomitant gene expression representing M1 macrophages, T follicular helper cells, and B cells was observed in this same subgroup, while the remaining tumors showed low immune infiltration.

**Fig. 4:**
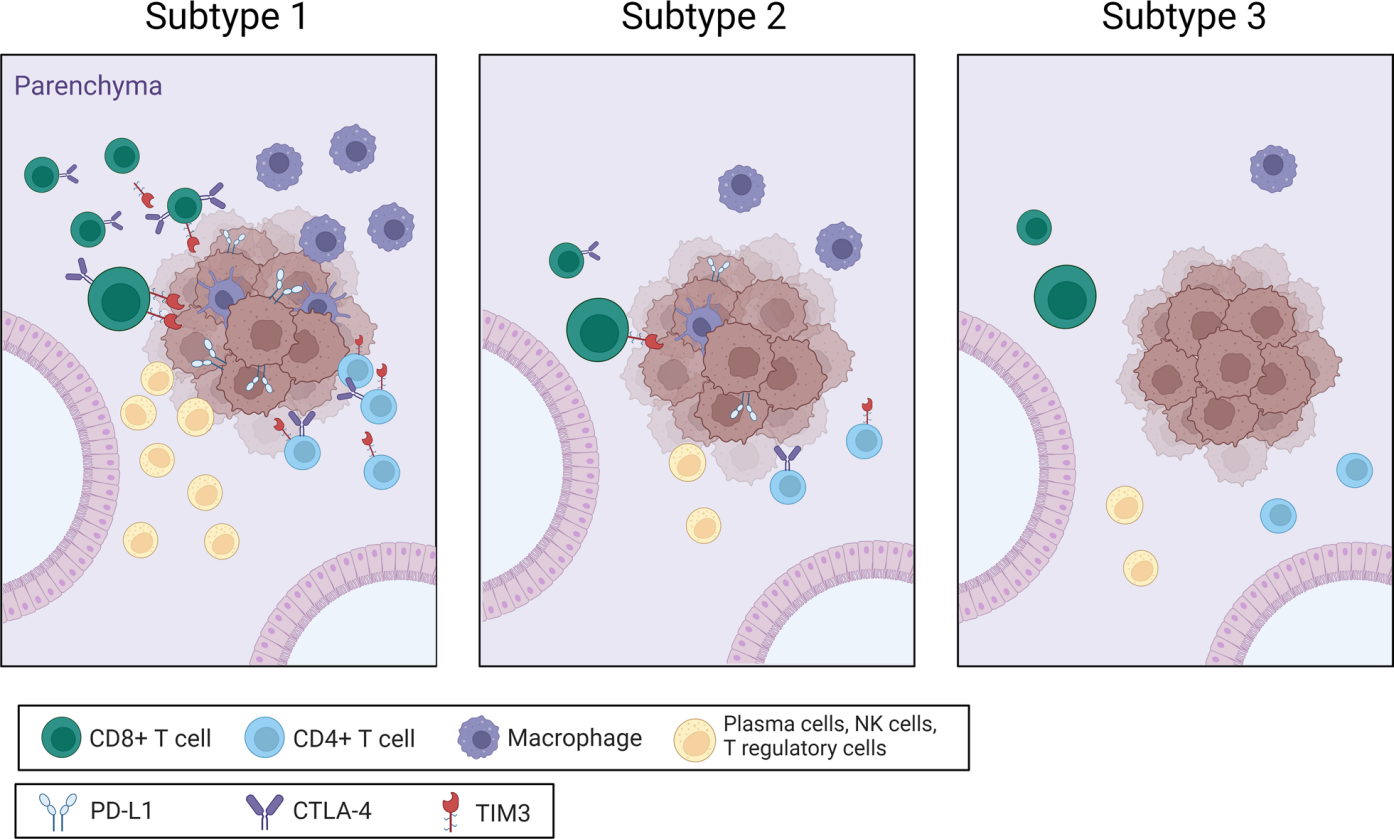
The spectrum of LCNS tumor immune environments as determined by RNA-Seq. Subtype 1 has high immune cell abundance and high immune marker expression. Subtype 2 has significantly lower immune cell levels and relatively lower immune cell marker expression. Subtype 3 has similarly low immune cell populations as the subtype 2, with the exception of macrophage levels decreased, and immune marker expression is significantly downregulated [20].

This gradient of immune cell type abundance was also reported in Devarakonda et al., whose cohort separated evenly into three immune subtypes. The first subtype had high expression of immune markers like PD-L1, TIM3, and CTLA4 and the highest numbers of every immune cell type. The second subtype contained mixed immune cell frequencies and immune checkpoint molecules that were overall lower than those of the first subtype and the third subtype was relatively depleted on both fronts (Fig. 4). The diversity in immune activity between LCNS tumors identifies a potential subgroup of patients who may be more responsive to immune checkpoint inhibitor therapies. However, identification of patients with immunologically ‘hot’ tumors remains difficult as neither KRAS mutations, EGFR mutations, nor TMB were significantly different amongst subtypes [20]. Further investigation of how LCNS patients with different immune profiles respond to immune checkpoint inhibitors, as well as other systemic therapies, can guide more personalized treatment strategies and provide important information about prognosis.

## Implications for clinical management

### Screening and early detection

LCNS patients are often diagnosed at later stages due to the lack of suspicion of lung cancer in the never smoking population, resulting in poorer clinical outcomes [56]. The methods employed in large-scale genomic studies of tumor samples allow insight into the carcinogenesis of LCNS and thus possibilities for prevention and earlier detection. Namely, these aforementioned studies have been able to detect risk factors that may help to identify a population of never smokers who are contenders for screening and to propose biomarkers that can aid in evaluating risk of LCNS at an individual level.

#### Epidemiological risk factors

Identifying a target population for screening and early detection of LCNS remains an unanswered question, particularly taking into consideration that never smokers represent a large group that would not be cost-effective to screen at a population-wide level. Environmental risk factors such as exposure to outdoor air pollution, diet, and second-hand smoke are three potential etiologic agents that have been identified through tumor biology as risk factors that may aid in this endeavor.

A mutational signature indicative of air pollution exposure was present in all three cohorts. Nitro-PAH is a product of incomplete combustion that exists primarily as fine particles [57]. Commonly present as particulate matter smaller than 2.5 μm (PM_2.5_), PAHs and nitro-PAHs pose a threat to lung health as they can be inhaled into the small airways and alveoli and accumulate over time [58]. A previous meta-analysis determined that every 10 μg/m [3] incremental increase in PM_2.5_ exposure significantly increases relative risk of lung cancer in never smokers [59]. While only present in a small percentage of patients, the common findings of air pollution signatures by Chen et al., Devarakonda et al., and Zhang et al. provide biological evidence that air pollution constituents have an imprint on the cancer genome among never smoker lung cancer patients and may suggest that air pollution exposure is a robust predictor of risk for LCNS across varying ethnicities and geographical locations.

Only the Taiwanese cohort possessed two distinct mutational signatures, those representing nitro-PAH and DBAC exposure, that are connected to air pollution. This is potentially owing to total concentrations of PM_2.5_ that in recent years are up to four times higher in East Asia than those of North America and European countries [60]. Due to also a higher number of patients harboring the nitro-PAH signature in the Taiwanese cohort, subsequent subgroup analysis was conducted and found older females to be overrepresented, suggesting that increased exposure time likely increases risk but also questioning if females are more susceptible to air pollution exposure [21]. Recent pre-clinical and clinical research have revealed that females have greater inflammatory response to ambient air pollution than males, so this is an area of future investigation for purposes of risk calculation [61, 62].

The significance of PAH and nitro-PAH signatures present in LCNS tumors is that it supports the use of air pollution exposure as a lung cancer risk factor in never smokers. This can be translated into the clinic by screening for PM_2.5_ exposure, which has been effectively conducted in Myers et al., who correlated long-term address of residence with satellite PM_2.5_ data and found that PM_2.5_ exposure is an independent risk factor for LCNS [63]. The social implications of these findings can also be appreciated, as those of lower socioeconomic status tend to live in areas of greater fine particle pollution and may have less access to healthcare services. Therefore, an additional area of future focus may be on how to reach these at-risk populations.

Another source of nitro-PAH is biomass burning [45]. A 20-year cohort study recently reported an increased incidence of lung cancer in those who had long term exposure to wildfire smoke [64]. In conjunction with the increasing frequency of forest fires in recent decades in the West of North America [65], this may warrant further investigation of LCNS risk for populations who live in close proximity to forest fire prone areas. In the future, similar to ambient air pollution exposure, wildfire smoke exposure may be screened for based on address of residence for estimation of LCNS risk. This can be conducted using satellite and ground data as modelled previously modelled for fine particulate matter in a study by Myers et al. [63].

A nitrosamine mutational signature noted in Chen et al. represents the first record of diet potentially impacting the genome of LCNS patients. Commonly found in processed meats due to the use of its precursor nitrate as a preservative, nitrosamines present in high concentrations within bacon, sausage, and other cured foods [50]. In addition, specific types of nitrosamines are also found in chewing tobacco, tobacco smoke, salt-preserved fish and vegetables, and alcohol, specifically beer [50, 66]. Nitrosamine intake has been linked to gastric cancer and specific nitrosamine compounds have been recognized as carcinogens by the International Agency for Research on Cancer [67, 68]. These foods are more common in the East Asian diet than the Western diet [69], which may elucidate why nitrosamine mutational signatures were only present in the Taiwanese LCNS cohort and not in the primarily Caucasian cohorts.

Although difficult to incorporate into a screening protocol due to the variability and non-specificity of diet, this suggests that a diet that limits processed meat, smoked food, and alcoholic beverage intake may be a possible protective factor from LCNS.

Finally, while it is possible that SHS plays a role in LCNS, there is currently a lack of genomic evidence that supports it as a strong factor that drives carcinogenesis. Across the three LCNS cohorts, there were limited to no tobacco related mutational signatures. However, only Zhang et al. asked patients for their SHS exposure history and in general it is a difficult factor to measure. It can also be difficult to distinguish between SHS and other smoke- or pollution-related exposures where tobacco is not involved. PAHs, for example, are present in SHS as well as diesel exhaust and overall ambient air pollution [70]. This reiterates that the combustive nature of SHS may contribute to LCNS carcinogenesis but may only drive disease in a small number of patients and likely requires additional risk factors to cause lung cancer. Thus, when SHS exposure is inquired about on history, it must also be considered in the context of other risk factors.

#### Biomarkers for risk assessment

LUAD is an aggressive cancer type that can metastasize quickly and there is a need to identify reliable biomarkers that allow high risk patients to be identified for surveillance in hopes of early detection and treatment initiation [71]. Zhang et al. found a median latency period of eight years between the emergence of an EGFR-mutated tumor’s MRCA and the tumor becoming clinically detectable. This provides a window for screening that would allow for early detection of LCNS. Molecular testing for NSCLC patients in all stages is the current standard of care [in Canada] and involves PCR-based methods on biopsy samples, which is too invasive for screening purposes [72]. However, the use of plasma-derived cell-free DNA may is a potential future option to screen and monitor EGFR mutations [73]. It should be noted that this approach is still in its infancy as it has shown poor sensitivity to date [74], and it is currently only approved for finding EGFR T790M resistance in patients with diagnosed NSCLC. Further research in this area may supply a method to screen for high risk patients—such as those who have known high air pollution exposure—who can be followed up closely for lung cancer development or who also qualify for screening through low-dose CT, which is presently only available for current and former smokers [75, 76].

Germline variants contribute to LCNS carcinogenesis; two of the three LCNS tumor cohorts investigated pathogenic germline variants (PVs) and their presence was detected in 6.9% to 36.6% of LCNS tumors [21, 22]. Although the penetrance and relevance of each PV varies, a recent study found moderate to high penetrance PVs in 4.3% of a 5118 NSCLC cohort of varied smoking statuses, and this correlated with family history of any cancer as well as age of diagnosis before the age of 55 [77]. A multi-center cohort study has previously found that risk of lung cancer is 51% higher in never smoker females with one first degree relative with lung cancer and 123% with two relatives affected [78]. These findings highlight the importance of evaluating personal and family cancer history with consideration to refer high-risk patients with LCNS to hereditary cancer services for germline testing if available. While there are no clear guidelines for lung cancer screening in never smokers, proactive surveillance is imperative as these patients tend to present at younger ages [79].

Some cancer germline variants were exclusive to never smokers with lung cancer, suggesting that further research to curate a list of LCNS associated PVs and their penetrance may be clinically relevant for calculation of a combined lung cancer risk score for never smoker patients. Both Zhang et al. and Devarakonda et al. found BRCA1 to be a PV exclusive to never smoker patients, which has been shown to be associated with lung cancer, specifically LUAD [80]. In addition, germline mutations in mismatch repair gene MSH6, which was present in both cohorts, has previously been found to be present in 1% of lung cancer patients [81]. Further research may unveil if MSH6 is preferentially altered in LCNS patients. Other germline modified genes associated with lung cancer that were identified include ATM, CDK4, FANCM, and POLD1 [82]. Findings from GWAS studies have recently been leveraged to create polygenic risk score (PRS) models for LUAD, with one study’s PRS more strongly correlating with LCNS [54, 83]. Another study investigating Taiwanese female LCNS patients found reliable prediction of 6-year incidence risk in a model that used GWAS data in combination with clinical factors [84]. There are significant variations in the genes and loci employed in these models and a curated list of approved genetic factors have yet to be employed in the clinic. However, testing for PVs and high-risk loci may offer a valuable, non-invasive avenue for risk assessment and early detection of lung cancer, particularly for never smokers who have other risk factors, including a known family history of cancer. [85].

### Treatment

The treatment of LCNS varies greatly from that of lung cancer in smokers and the majority of patients present with advanced stage disease at diagnosis [86]. A significant proportion of LCNS patients are eligible for targeted therapies; however, patients ultimately develop resistant disease [87]. Moreover, a significant proportion of LCNS patients have no targetable driver mutations, representing a subgroup with poorer clinical outcomes as compared to patients with targetable driver mutations [88]. Therefore, it is vital that the genomic characterization of LCNS tumors is correlated with treatment responses and clinical outcomes to not only inform therapy selection, but also to advance discovery of targetable driver mutations and novel therapies.

#### Driver gene mutations and targeted therapy

As compared to smokers with lung cancer, EGFR mutations occur at a high frequency among LCNS patients, the majority of which can be treated with oral targeted EGFR tyrosine kinase inhibitors (TKIs), osimertinib, as a first line treatment for such patients in advanced stages of disease [89]. Among a Caucasian cohort from Zhang et al. and a Taiwanese cohort from Chen et al., 30.6% and 85% of patients harbored EGFR mutations, respectively.

EGFR-mutated lung cancer patients who are former or current smokers have shorter progression free survival (PFS) and overall survival compared to never smokers. Furthermore, as total pack year history increases, PFS decreases across treatment regimens [31, 90]. This suggests a spectrum of responses between smoking and never smoking tumors even when a driver mutation is identified. Among the two common EGFR mutation subtypes, exon 19 deletion is associated with longer PFS than exon 21 L858R substitution in NSCLC patients [91]. The proportion of LUAD patients harboring either EGFR mutation subtype did not seem to differ between ethnicities in LCNS nor compared to smokers from independent studies although this data has not been formally analyzed [20, 21, 31]. Consideration of EGFR mutation subtypes and their clinical outcomes may thus inform prognosis and clinical management.

Presence of a tumor TP53 mutation is a negative prognostic factor and the subgroup of EGFR mutated LCNS patients with TP53 co-mutations are observed to have poorer outcomes with targeted therapies and are more likely to develop resistance to therapy [92, 93]. However, TP53 and EGFR co-mutations were observed in tumors with higher SCNAs and TMB, which are positive predictors for response to immunotherapy [22]. TP53 mutated NSCLC patients have shown benefit from immunotherapy and while never smoker patients have been shown to have poor response to immunotherapy [94], further studies of patients with co-mutations are needed to evaluate whether there is a role for immunotherapy either alone or in combination with chemotherapy and/or targeted therapy for this subgroup.

EGFR mutations are also observed to occur frequently with CDKN2A and RB1 mutations in LCNS tumors, with loss of function variants identified in CDKN2A and RB1 at 24.4% and 16%, respectively; EGFR-positive lung cancers harboring co-mutations have been associated with increased resistance to EGFR-TKI therapy and poorer clinical outcomes than harboring EGFR mutation alone [95]. There are preclinical data suggesting that the combination of EGFR-TKI and CDK4/6 inhibitors may therefore be beneficial in co-mutated EGFR and CDKN2A patients as it may prevent gain of resistance against TKIs [96]. CDK4/6 inhibitors are not approved for use in NSCLC but they have shown promise in vitro against EGFR-TKI resistant lines and are currently being studied as combination therapy in early-stage clinical trials [96–98]. To date, no RB1 related therapies are currently under investigation for NSCLC, but RB1 been shown as a negative prognostic factor when co-occurring with both TP53 and EGFR mutation and is significantly more susceptible to histological transformation to small cell lung cancer [99]. The awareness of EGFR co-mutations can thus potentially inform patient surveillance, management, and prognosis.

Despite EGFR mutation being a common somatic alteration in LCNS, a significant proportion of LCNS patients harbor other driver mutations or none at all, particularly among Caucasian patients [22]. Considering that non-EGFR actionable molecular alterations are present in up to 20% of LCNS, it is crucial to rule out such modifications before considering other treatment avenues [22, 100]. Zhang et al. found that EGFR mutation negative tumors generally have low TMB and structural alterations [22], thus these cases may be more amenable to relevant cytotoxic therapy as compared to immunotherapy. This tumor subtype includes 76.5% of KRAS mutated cases and all of the tumors that harbored RET fusions within the Zhang et al. cohort [22]. However, LCNS patients are more likely to have KRAS G12D mutation as opposed to KRAS G12C, which is the only KRAS mutation that is targetable with currently approved therapy [101, 102]. RET fusion is a rare molecular alteration in lung cancers although its prevalence is twice as high in never smokers [103]. Recent clinical trials have led to the approval of use of targeted therapies including pralsetinib and selpercatinib for RET-fusion positive advanced NSCLC, thus these serve as first-line treatment options for the select LCNS patients with low TMB and lack structural modifications but harbor RET fusion [104, 105].

Regarding the remaining tumors with no known driver mutation identified, genes that contribute to their stem-like state may serve as therapeutic targets, such as NOTCH1 and ARID1A. Interestingly, in a retrospective study on anti-PD-1 treated cancer patients including those with NSCLC, responders tended to have higher prevalence of mutations in NOTCH1 and ARID1A relative to non-responders [106]. These data support further studies to evaluate the potential role for immunotherapy as a treatment strategy for a specific subset of LCNS patients lacking targetable driver mutations.

#### Mutational processes and novel therapeutic approaches

Mutational profiling can have powerful implications to inform therapy selection and prognosis determination. APOBEC signatures were observed across all LCNS cohorts, confirming previous identification of these signatures in both smoking and never smoking lung cancer [107, 108]. Chen et al. found that APOBEC signatures were more abundant in younger females and were also present in 100% of females without an EGFR mutation. Along the same vein, those who lacked APOBEC signatures almost always had an EGFR mutation [21]. It has been shown that high APOBEC signature is associated with better PFS in advanced NSCLC that has been treated with immunotherapy, namely PD-L1 inhibitors [109]. This may suggest that younger EGFR wild type female patients have better responses to immunotherapy. Similarly, Chen et al. also showed that APOBEC3 proteins were present at higher levels in females and those with higher APOBEC3 protein expression had high expression of kinases like CK2 and CDK1. This brings forth the question of whether inhibition of these proteins may be an effective future neoadjuvant treatment strategy for these patients, as has been suggested by preclinical studies [110, 111]. In contrast, Zhang et al. and previous studies have found APOBEC signatures to be highly correlated to EGFR mutation and not dependent on sex, although this may be due to a lack of distinction between APOBEC3 proteins, lack of subgroup analyses, ethnicity differences, or otherwise [21]. A future direction would therefore be to explore the LCNS tumor genomic landscape further while stratifying by factors such as age and sex.

Other mutational signatures that have been identified within LCNS tumors vary with cancer evolution; for example, SBS1 and SBS5 generally present in earlier stage cancers whereas presence of SBS18—which represents DNA damage from ROS—increases in later stages [37]. SBS1 has been shown to correlate with lower immune infiltration in LUAD [37], and thus could have predictive power regarding immunotherapy response. Additionally, tumors harboring SBS5 have been associated with better prognosis in NSCLC patients [37]. Despite not being previously studied in NSCLC, SBS18 has been associated with tumor metastasis and worse prognosis in breast and prostate cancer [112, 113] and it has been strongly linked with sensitivity to EGFR inhibition to afatinib [114]. Thus, mutational signatures can offer unique information that is distinct from that of molecular drivers and gene expression and investigating the relationship between mutational signatures and LCNS evolution may allow for better patient management in the future.

#### Immunotherapy

At the genomic and transcriptomic level, immune profile was observed to be on a spectrum within each of the LCNS tumor cohorts, ranging in the presence and proportions of various immune cell types and the degree of their immune checkpoint molecule expression including PD-L1, TIM-3, and CTLA4 [20]. LCNS tumors generally respond poorly to immunotherapy compared to those of ever smokers [115]. Even in the context of high PD-L1 expression across patients, previous research has found that anti-PD-1 monotherapy had lower overall response rates and one year survival rates in never smokers than smokers [116]. This has been hypothesized to be due to the overall lower TMB in never smokers, as TMB has been emerging as a positive predictive marker for anti-PD-1 therapy. Thus, for subsequent-line treatments or if targeted therapy is not an option, chemotherapy is generally be considered before immunotherapy in never smokers due to better response [117]. However, there is an exception among EGFR wild type patients, who have higher response rates with immunotherapy as compared to those with EGFR or ALK driver mutations. Thus, immunotherapy with or without chemotherapy are standard treatment options for EGFR and ALK negative NSCLC patients regardless of smoking status [118]. This may be related to the association of EGFR wild type tumors being more likely to harbor APOBEC signatures, as observed by Chen et al. More studies evaluating the relationship of EGFR status and mutational signatures with treatment responses in the context of LCNS are needed to potentially inform treatment for never smoking patients with no targetable driver mutations.

Another study by Chen et al. found that immune related proteins were highly expressed in stage IA tumors, suggesting that immune signalling to be most active in early stages of LCNS and thus may contribute to why response to immunotherapy is limited in later stage LCNS [119]. The heterogeneity of immune response between LCNS tumors likely also plays a role in the overall lack of response to immune-targeted therapies and suggests that particular subgroups within the LCNS population may confer benefit over others [115]. However, it should be noted that immune cell levels pre-treatment are not always predictive of response to immunotherapy [120], which calls for further research into predictors of immune response to treatment. In the future, the sequencing of biopsy tissue combined with other methods of immune profile characterization may help to identify promising candidates for immunotherapy, particularly when other options are limited for the patient.

## Conclusion

Integrative analyses of large LCNS patient cohorts have provided rich insight into the clinical presentation and pathogenesis of LCNS. These results can be leveraged in the form of screening and early detection that can potentially incorporate factors such as air pollution exposure, SHS exposure, family history, and possibly even diet to stratify lung cancer risk in never smokers. The molecular status of a LCNS tumor can be critical in treatment selection, particularly due to the high number of targetable mutations present within LCNS tumors. Findings from ongoing studies of LCNS tumor biology including mutational signatures, protein expression, and immune profiles also have potential to further inform prevention, screening, diagnosis, and identify novel therapeutic approaches for this difficult to treat disease.

## Data Availability

Not applicable.

## Abbreviations

ALK: Anaplastic lymphoma kinase
APOBEC: Apolipoprotein B mRNA editing catalytic polypeptide
AR: Androgen receptor
ARID1A: AT-rich interaction domain 1A
ATM: Ataxia-telangiectasia mutated gene
BRCA1: Breast cancer gene 1
CDK1: Cyclin dependent kinase 1
CDK2: Cyclin dependent kinase 2
CDKN2A: Cyclin dependent kinase inhibitor 2A
CK2: Casein kinase 2
COPD: Chronic obstructive pulmonary disease
COSMIC: Catalogue of somatic mutations in cancer
CTLA4: Cytotoxic T-lymphocyte associated antigen 4
CYP21A2: Cytochrome P450 family 21 subfamily A member 2
DNA: Deoxyribonucleic acid
EGFR: Epidermal growth factor receptor
FANCG: Fanconi anemia complementation group G gene
GWAS: Genome-wide associated study
KRAS: Kirsten rat sarcoma viral oncogene homolog
LCNS: Lung cancer in never smokers
LUAD: Lung adenocarcinoma
MRCA: Most recent common ancestor
MSH6: MutS homolog 6
NF1: Neurofibromatosis type 1
Nitro-PAH: Nitrated polycyclic aromatic hydrocarbon
NKX2-1: Homeodomain NK2 homeobox 1
NOTCH1: Neurogenic locus notch homolog protein 1
NSCLC: Non-small cell lung cancer
PAH: Polycyclic aromatic hydrocarbon
PD-L1: Programmed death ligand 1
PFS: Progression free survival
PM2.5: Particulate matter smaller than 2.5 µm
POLD1: Polymerase delta 1
PRS: Polygenic risk score
PV: Pathogenic germline variants
RB1: Retinoblastoma 1
RET: Rearranged during transfection
RNA: Ribonucleic acid
ROS: Reactive oxygen species
RTK/RAS/RAF: Receptor tyrosine kinase/rat sarcoma protein/rapidly accelerated fibrosarcoma protein
SBS: Single base substitution
SCNA: Somatic copy number alteration
SHS: Second hand smoke
TIM3: T cell immunoglobulin and mucin domain 3
TKI: Tyrosine kinase inhibitor
TMB: Tumor mutational burden
TMEM127: Transmembrane protein 127
TP53: Tumor protein 53
UV: Ultraviolet
